# The Separation of Antler Polypeptide and Its Effects on the Proliferation and Osteogenetic Differentiation of Bone Marrow Mesenchymal Stem Cells

**DOI:** 10.1155/2020/1294151

**Published:** 2020-09-30

**Authors:** Ping Wang, Tie-Feng Sun, Gang Li, Hui-Min Zhang, Fan-jie Liu, Zhi-hui Gao, Sheng-nan Cao, Guo-dong Sun, Hai-tao Du, Cong-an Wang, Dan-dan Wang, Bin Shi, Ling Lin

**Affiliations:** ^1^State Key Laboratory of Precision Measurement Technology and Instruments, Tianjin University, Tianjin 300072, China; ^2^Shandong Academy of Chinese Medicine, Jinan 250014, China; ^3^Bone Biomechanics Engineering Laboratory of Shandong Province, Key Laboratory for Biotech-Drugs of National Health Commission, Neck-Shoulder and Lumbocrural Pain Hospital of Shandong First Medical University, Shandong Medicinal Biotechnology Center, Shandong First Medical University and Shandong Academy of Medical Sciences, Jinan 250062, China; ^4^School of Pharmaceutical Sciences, Shandong University of Traditional Chinese Medicine, Jinan 250355, China; ^5^Department of Traditional Chinese Medicine Orthopedics, The Third Affiliated Hospital of Shandong First Medical University, Shandong First Medical University and Shandong Academy of Medical Sciences, Jinan 250062, China

## Abstract

**Background:**

Colla Cornus Cervi (CCC) has been used as a traditional Chinese medicine in the treatment of osteoporosis and osteonecrosis of the femoral head. However, the bioavailability of CCC is seriously limited owing to its large molecular weight and complex ingredients. In the present study, antler polypeptide was separated from CCC, and the effects of antler polypeptide on rat bone marrow mesenchymal stem cells (BMSCs) were investigated.

**Methods:**

Antler polypeptide was separated from Colla Cornus Cervi by ultrafiltration into different samples according to the molecular weight. The total peptide content of these samples was determined by the biuret method. The content of antler polypeptide in different samples was quantified by high-performance liquid chromatography (HPLC). The effects of antler polypeptide at different concentrations on the proliferation, cell cycle, alkaline phosphatase activity, and BMP7 expression of BMSCs were investigated.

**Results:**

Antler polypeptide was separated by ultrafiltration into different samples: A (molecular weight <800 Da), B (molecular weight 800–1500 Da), and C (molecular weight >1500 Da). The total peptide contents of A, B, and C were 0.602 mg/mL, 8.976 mg/mL, and 38.88 mg/mL. Antler polypeptide B eluted at 14.279∼15.351 min showed that the content of antler polypeptide was significantly higher than that of A and C with a peak area of 933.80927. The BMSCs proliferation rate (84.66%) of polypeptide B was the highest at the concentration of 1.578 × 10^−2^ g/mL. Antler polypeptide B significantly promoted the proliferation of BMSCs with a proliferation index of 38.68%, which was significantly higher than that of the other groups. Antler polypeptide B significantly enhanced the activity of alkaline phosphatase in BMSCs compared to that of the blank group (*P* < 0.001). Antler polypeptide B increased the BMP7 protein expression in BMSCs.

**Conclusions:**

Results suggested that antler polypeptide may promote the proliferation and osteogenic differentiation of BMSCs. Our study lays an experimental foundation for the further development and application of antler polypeptide in medicine.

## 1. Introduction

Avascular Necrosis of the Femoral Head (ANFH) refers to the pathological process of necrosis of bone cells, bone marrow hematopoietic cells, and adipocytes caused by partial or complete ischemia of the femoral head due to different reasons. With the aging of social population, ANFH has been listed as one of the three major geriatric diseases by WHO, and the prevalence rate has increased year by year. The treatment of ANFH is closely related to bone regeneration. We noticed the specificity of antlers in the study of bone regeneration. The once-a-year regeneration cycle of antlers is a specific cut regeneration and rapid growth model of mammalian with the feature of rapid coordinated regeneration of nerves, blood vessels, hoof tissues, cartilage, skin, and bone [1]. Especially, the polypeptide composition of Colla Cornus Cervi (CCC) extracted from antler is similar to that of bone glue fiber, and CCC contains much nutrients and cytokines that promote bone growth. We hope to find the possible way of promoting bone growth and regeneration from antler polypeptides.

CCC, the main extract of antlers, has been used as a traditional Chinese medicine for nearly two thousand years. The *Commentaries on the Illustrations* records that “Old deer has good antlers. Boiling antlers to obtain Colla Cornus Cervi, which is better to use as medicine” [2]. According to Chinese Pharmacopoeia, CCC is a solid glue made from the decoction in which antler slices are boiled and concentrated, and the antler slices are the ossified antlers of red deer (*Cervus elaphus* Linnaeus) or sika deer (*Cervus nippon* Temminck) or their antler bases fallen off in the following spring after sawing antler [3]. Modern studies have shown that CCC is effective in anti-inflammation and analgesia, protecting of gastric mucosa, antimammary gland hyperplasia, improving sexual function, brain protection, and preventing osteoporosis [4–6]. CCC is full of protein, accounting for more than 82.49% of all ingredients [7]. It is not only the material foundation of the efficacy of antler gum but also the key which decides the quality of antler gum [8]. CCC has been reported to be effective in the treatment of osteoporosis and osteonecrosis of the femoral head [7]. This is also corresponding to the effect of deer horn glue recorded in Chinese medical classics on strengthening kidney yang, producing essence and to unifying marrow, and strengthening muscles and bones [4]. A previous study revealed that CCC promoted the proliferation and osteogenic differentiation of bone marrow-derived mesenchymal stem cells (BMSCs), and CCC and BMP7 play an important role in the ability of BMSCs to facilitate repair of avascular necrosis of the femoral head (ANFH) in rats [9]. However, the utilization and bioavailability of CCC is seriously limited owing to its large molecular weight and complex ingredients [10]. Thus, it is important to improve the bioavailability of CCC in order to fully exploit this valuable Chinese medicine resource to better help the improvement of human health.

In the recent years, more and more studies have focused on the physiological functions of peptides, especially gum medicines whose main active components are proteins that are difficult to absorb directly [11]. Antler polypeptide, which is isolated from antlers with a molecular weight between 0.2 and 10 kDa, takes part in many biological functions and multiple activities [12]. Due to its small molecular weight, simple structures, low immunogenicity, and easy absorption and utilization by cells, antler polypeptide has been widely used clinically to provide nutrition for the human body [13–15].

In the present study, we prepared an enzymatic hydrolysate of antler polypeptide by a previously reported method [16] and separated the lysate into three parts according to relative molecular mass by ultrafiltration. Then, high-performance liquid chromatography (HPLC) and the biuret method were used to quantify antler polypeptide in different samples. The effects of different concentrations of the antler polypeptide solutions on the proliferation, cell cycle, and osteogenic differentiation of BMSCs were investigated. Our study lays an experimental foundation for the further development and application of antler polypeptide in medicine.

## 2. Materials and Methods

### 2.1. Materials

CCC is solid glue made from the decoction in which antler slices are boiled and concentrated. Antler tablets (batch number 150470, Hebei Yabao Pharmaceutical Co. Ltd., China) were identified as originating from red deer by the Chinese Medicine Research Institute of Shandong Academy of Traditional Chinese Medicine. Preparation and identification were carried out according to the 2020 edition of the Chinese Pharmacopoeia section on CCC [3].

Pepsin (Meilun Bio, China), Dulbecco's modified Eagle's medium-low glucose (DMEM-LG, Hyclone Co. Ltd., USA), 0.25% trypsin-ethylenediamine tetraacetic acid solution (Solarbio Co. Ltd., China), penicillin and streptomycin mixture (Solarbio Co. Ltd., China), phosphate buffered saline (PBS, Solarbio Co. Ltd., China), bovine serum albumin (BSA, purity 98%, Solarbio Co. Ltd., China), fetal bovine serum (FBS, Tianjin Haoyang Biological Products Technology Co. Ltd., China), Cytochrome C (Mr 12,500 Da), aprotinin (Mr 6,500 Da), bacillus enzyme (Mr 1450 Da), glycine-glycine-tyrosine-arginine (Mr 451 Da), glycine-glycine-glycine (Mr 189 Da), and BMSCs were purchased from Saiye Biotechnology (article number RASMX-01001, Guangzhou, China). A rabbit polyclonal antibody against BMP-7 (Abcam, Britain), a rabbit polyclonal antibody against GAPDH (Santa Cruz, USA), and a horseradish peroxidase- (HRP-) conjugated goat anti-rabbit IgG antibody (Santa Cruz, USA) were used.

### 2.2. Instruments

A microfiltration-ultrafiltration-nanofiltration membrane separation tester (BONA-GM-18, Jinan Bona Biotechnology Co. Ltd., China), ZX-002 UV spectrophotometer (Shimadzu, Japan), Waters 2965 HPLC apparatus (Waters Co. Ltd., USA), KDM-type temperature control electric heating mantle (Hualuyiqi Co. Ltd., China), BP211D Sartorius electronic analytical balance (Germany Sartorius Co. Ltd., Germany), HH-S6 digital constant temperature water bath (Jintan Medical Instrument Factory, China), LGJ-10 freeze dryer (Shengchao Kechuang Biotechnology Co. Ltd., China), −80°C refrigerator (FOMAS Co. Ltd., USA), plastic culture plates (Corning Co. Ltd.), inverted phase contrast microscope (Olympus Co. Ltd., Japan), CO_2_ incubator (Changsha Huasheng Electronic Technology Co. Ltd., China), microplate reader (Bio-Rad Co. Ltd., USA), and ultraclean workbench (ZHJH-1209, Taiwan Sitea Equipment Co. Ltd., Taiwan) were used.

### 2.3. Preparation of Antler Polypeptide

The CCC powder and pepsin (4%, m/V) were placed in a 50 mL conical flask, and the pH was adjusted to the required level with acetic acid and sodium acetate buffer solutions. The solids were dissolved in water under ultrasonication and enzymolysis in a constant temperature water bath at enzymatic hydrolysis temperature of 40°C. After enzymatic hydrolysis, the enzyme was inactivated by heating to 95°C for 5 min. The mixture was centrifuged at 7000 g·min-1 for 10 min, and the supernatant was used to obtain the antler polypeptide hydrolysate.

### 2.4. Ultrafiltration Separation of Antler Polypeptide

The antler polypeptide hydrolysate was placed in an ultrafiltration system, and Hollow fiber ultrafiltration membranes with molecular weight of 800 and 1500 Da were used for ultrafiltration [17, 18]. The ultrafiltration operating temperature was 22–25°C, and the operating pressure was <0.1 MPa. The antler polypeptide was divided into antler polypeptide A (<800 Da), B (800–1500 Da), and C (>1500 Da). The samples were sterilized by cobalt 60 irradiation and stored at −20°C.

### 2.5. Determination of the Antler Polypeptide Content by the Biuret Method

Standard substance preparation: Standard BSA was diluted into a standard protein solution of 10.8 mg mL^−1^ using distilled water. Preparation of the biuret reagent: 1.50 g copper sulfate (CuSO_4_·5H_2_O) and 6.0 g sodium potassium tartrate (KNaC_4_H_4_O_6_·4H_2_O) were dissolved in 500 mL distilled water and 300 mL of 10% NaOH solution was added and diluted to 1 000 mL with distilled water. Standard curve: 12 tubes were taken into two groups, 0, 0.2, 0.4, 0.6, 0.8, and 1.0 mL standard BSA solution was added, it was complemented to 1 mL with distilled water, and then, 4 mL of the biuret reagent was added and placed at room temperature (20∼25°C) for 30 min. An absorbance value at 540 nm was determination with a microplate reader. Taking the concentration as the abscissa and the absorbance as the ordinate, the standard curve was obtained: *y* = 0.0464*x*−0.0022, *R*^2^ = 0.9998. The absorbance of antler polypeptide samples was determined using the same method, and each sample was performed in triplicate. According to the standard curve, the antler polypeptide content was calculated as follows:

(1) $$\begin{array}{c} P=\frac{\left(A+0.0022\right)*N}{0.0464}, \end{array}$$

*A*: absorbance value, *N*: the dilution rate, and *P*: the content of antler polypeptide.

### 2.6. Determination of the Antler Polypeptide Content by HPLC

The reference substances (cytochrome C, aprotinin, bacillus enzyme, glycine-glycine-tyrosine-arginine, and glycine-glycine-glycine) were accurately weighed. The mobile phase was configured using 0.1% (mass fraction) peptide standard solutions of different relative molecular weights. Sample preparation was performed using 200 *μ*L antler polypeptide A, B, or C, which was diluted with pure water 10 times and filtered with a microporous membrane (0.2–0.5 *μ*m polytetrafluoroethylene or nylon filter membrane). The injection volume was 10 *μ*L. A TSKgel G2000 SWXL (300 mm × 7.8 mm) column was used for separation with a mobile phase of acetonitrile/water/trifluoroacetic acid (45 : 55 : 0.1, *v/v/v*). The UV detection wavelength was 220 nm, the flow rate was 0.5 mL·min^−1^, and the column temperature was 30°C.

To validate the method, its accuracy (six injections), repeatability (six mixed reference analyses), and stability (0, 2, 4, 6, 12, 24 h) were evaluated. The relative standard deviations (RSDs) of the determined peak areas were less than 3%, confirming the viability of the method. The chromatograms of the samples were analyzed by reference to the peak times of the five standard substances. Then, using gel-permeation chromatography (GPC) data processing software, the chromatographic data of the sample were substituted into the calibration curve equation to calculate the relative molecular weight of the peptide in the sample and its distribution range. The sum of the relative peak area percentages of the antler polypeptides was calculated by peak area normalization.

### 2.7. Culture of BMSCs

BMSCs were placed in a 25 cm^2^ flask and cultured in a complex medium at 37°C in a 5% CO_2_ incubator. The complex medium was DMEM-LG containing 20% FBS, 100 U/mL penicillin, and 100 *µ*g mL^−1^ streptomycin. The complex medium was changed every 3 days. The fourth generation of BMSCs were collected and washed three times with PBS. After digestion with 0.25% trypsin, single cell suspension was harvested for further experiment. BMSCs were identified by flow cytometry (CD29, CD44, and CD90 were positive with the expression rates >70%, whereas CD34, CD45, and CD11b were negative with the expression rates <5%).

### 2.8. Determination of Proliferation of BMSCs

After digestion, the BMSCs cell concentration was adjusted to 2 × 10^4^ mL^−1^. Cells were seeded in a 96-well plate at a density of 2 × 10^3^ cells per well and cultured at 37°C in a 5% CO_2_ incubator for 12 h. The supernatant was discarded, and 50 *μ*L antler polypeptide A, B, or C and 50 *μ*L medium were added, while 50 *μ*L PBS and 50 *μ*L medium were used as a blank group. After culturing for 24 h, 48 h, or 72 h, the supernatant was discarded and 100 *μ*L PBS and 10 *μ*L of 5 mg mL^−1^ 3-(4,5-dimethylthiazol-2-yl)-2,5-diphenyltetrazolium bromide (MTT) were added to each well. The plate was, then, incubated at 37°C in a 5% CO_2_ incubator for 4 h. Then, the supernatant was removed. 150 *μ*L DMSO was added to each well. The plate was oscillated for 10 min, and the absorbance value at a wavelength of 490 nm was detected using a microplate reader. The effects of antler polypeptide A, B, and C on the proliferation rate of BMSCs were calculated as follows: proliferation rate = average OD value of experimental group/average OD value of control group × 100%.

### 2.9. Cell Cycle Assay

Flow cytometry (propidium iodide staining) was used to investigate the BMSCs cell cycle. BMSCs were seeded into six-well plates at a density of 2 × 10^6^ per well, which were randomly divided into a blank group, CCC group, and antler polypeptide A group, antler polypeptide B group, and antler polypeptide C group. After culturing for 24 h, BMSCs were digested with trypsin and resuspended in ice-cold PBS at a density of 3 × 10^6^ mL^−1^ and incubated with RNase in a 37°C water bath for 30 min. The cells were subsequently incubated with propidium iodide at 4°C in the dark for 30–60 min and analyzed using a flow cytometer. The cell cycle of BMSCs was analyzed using software of ModFit Lt for mac V1.01.

### 2.10. Detection of the Activity of Alkaline Phosphatase (ALP)

The BMSCs cells were seeded in a six-well plate at a density of 5 × 10^5^ per well and divided into a blank group, CCC group, antler polypeptide A group, antler polypeptide B group, and antler polypeptide C group. After culturing for 48 h, the supernatant was discarded. Then, 250 *μ*L lysate was added into the six-well plate, and the cells were lysed for 30 min. The absorbance at 520 nm was detected using the ALP detection kit according to the manufacturer's instruction. Three independent experiments were performed.

### 2.11. Western Blot Analysis

The cells of each group were lysed in RIPA lysis buffer at 4°C for 15 min. The lysates were cleared by centrifugation (12,000 rpm) at 4°C for 30 min to collect total protein. About 20 *μ*g protein samples were, then, separated by electrophoresis in a 10% SDS (sodium dodecyl sulfate) polyacrylamide gel and transferred onto a polyvinylidene fluoride membrane. After blocking the nonspecific binding sites for 60 min with 5% nonfat milk, the membranes were incubated with a rabbit polyclonal antibody against BMP-7 (at 1 : 1000 dilution) at 4°C overnight. The membranes were, then, washed with TBST (tris-buffered saline with tween-20) three times at room temperature for 15 min. After washing, the target protein was probed with the horseradish peroxidase- (HRP-) conjugated goat anti-rabbit IgG antibody (at 1 : 2000 dilution) at 37°C for 1 h. After three washes, the membranes were developed by an enhanced chemiluminescence system. The protein levels were normalized with respect to the GAPDH protein level which was detected using a rabbit polyclonal antibody against GAPDH (at 1 : 5000 dilution).

### 2.12. Statistical Analysis

Statistical processing was performed using SPSS 19.0. The experimental data are expressed as $\bar{x}\pm s$. The least significant difference method was used for comparison between groups. *P* < 0.05 was considered statistically significant.

## 3. Results

### 3.1. Ultrafiltration Separation of Antler Polypeptide

After ultrafiltration, three antler polypeptide liquids with different colors were obtained. The solution of the antler polypeptide A (molecular weight <800 Da) was clear and transparent, antler polypeptide B (molecular weight 800–1500 Da) was light yellow, sticky, with no obvious odor, and antler polypeptide C (molecular weight >1500 Da) was black brown.

### 3.2. Determination of Antler Polypeptides by the Biuret Method

The concentration of A, B, and C samples were 6.319 mg/mL, 7.181 mg/mL, and 7.776 mg/mL. The total peptide content of A, B, and C samples determined by the biuret method was 0.602 mg/mL, 8.976 mg/mL, and 38.88 mg/mL. The results are shown in Table 1.

### 3.3. HPLC Analysis of Antler Polypeptide

**Figure 1:**
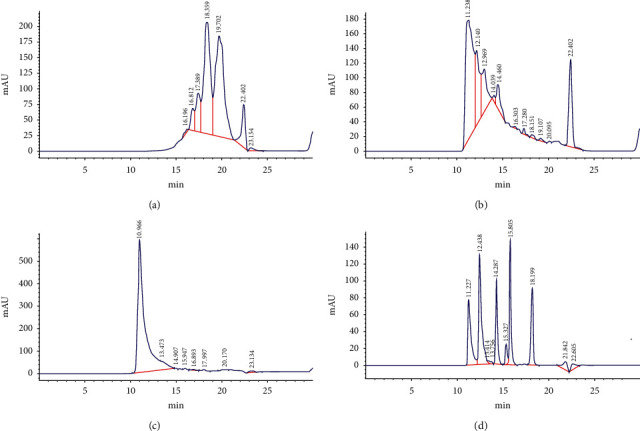
HPLC chromatograms of antler polypeptide solutions. Antler polypeptide A molecular weight <800 Da; antler polypeptide B molecular weight 800–1500 Da; antler polypeptide C molecular weight >1500 Da; peptide standard samples, 1, cytochrome C 2, aprotinin, 3, bacillus enzyme, 4, glycine-glycine-tyrosine-arginine, and 5, glycine-glycine-glycine. Elution time: 14.461–15.454 min.

The results are shown in Figure 1. The antler polypeptides with molecular weights <800 Da, 800–1500 Da, and >1500 Da were successfully separated. Comparing the retention times of the five standard materials (cytochrome C, aprotinin, bacillus enzyme, glycine-glycine-tyrosine-arginine, and glycine-glycine-glycine), the peptide peak (peak area 933.80927) with the molecular weight 800–1500 Da eluted at 14.279∼15.351 min showed that the content of antler polypeptide was significantly higher than that of the other samples (Table 2).

### 3.4. Morphological Observation of BMSCs

**Figure 2:**
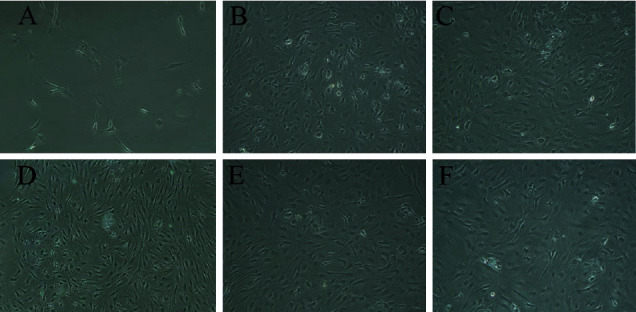
BMSCs observed under an inverted phase contrast microscope. (a) BMSCs cultured with a complex medium for the 2nd day. (b) BMSCs cultured with a complex medium for the 5th day. (c) BMSCs cultured with a complex medium containing antler polypeptide A for the 5th day. (d) BMSCs cultured with a complex medium containing antler polypeptide B for the 5th day. (e) BMSCs cultured with a complex medium containing antler polypeptide C for the 5th day. (f) BMSCs cultured with a complex medium containing Colla Cornus Cervi for the 5th day. Magnification × 200.

The morphology of the BMSCs was observed under an inverted phase contrast microscope (Figure 2). After two days of cell growth, the BMSCs showed single or multiple cell clones. On the day 5th, the BMSCs covered the bottom with a density of 90% and presented long wedge shapes.

### 3.5. Effects of Antler Polypeptide on the Proliferation of BMSCs

**Figure 3:**
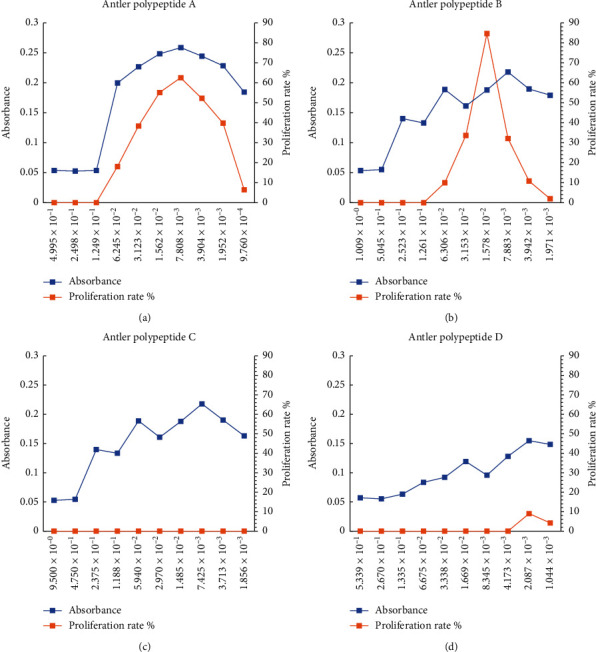
Effects of different concentrations of antler polypeptide on the proliferation of BMSCs (*n* = 4); the abscissa showed concentration (g/mL), primary ordinate showed absorbance, and minor ordinate showed the proliferation rate (%).

BMSCs were cultured with a complex medium containing different concentrations of antler polypeptides A, B, C, or CCC, respectively, to evaluate the proliferation of BMSCs. The results are shown in Figure 3. The antler polypeptides A, C, and CCC group take no obvious effect on the proliferation of BMSCs. Antler polypeptide B significantly promoted BMSCs proliferation, and the effect was the most positive when the concentration of antler polypeptide B was 1.578 × 10^−2^ g/mL. As the concentration decreased, the proliferation rate of BMSCs decreased.

### 3.6. Effects of Antler Polypeptide B on the Growth of BMSCs

**Figure 4:**
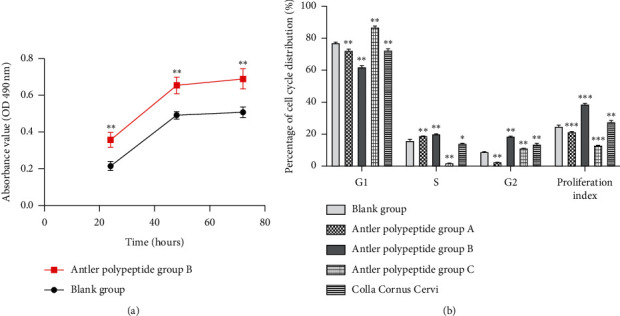
Antler polypeptide B promoted the growth of BMSCs. (a) Cell proliferation assay showed that antler polypeptide B promoted the proliferation of BMSCs (b) Effects of different antler polypeptide groups on the cell cycle of BMSCs. The proliferation index reflected the cell cycle process and proliferative ability of BMSCs. The antler polypeptide B group showed a significantly higher proliferation index. ^*∗*^*P* < 0.05, ^*∗∗*^*P* < 0.01, ^*∗∗∗*^*P* < 0.001, compared with the blank control group.

When BMSCs were cultured with a complex medium containing antler polypeptide B, the proliferation rate of BMSCs was the highest at 48 h and slower after 72 h (Figure 4(a)). The proliferative effects for both these time points were significantly better than that at 24 h. Compared with the blank group, antler polypeptide B significantly enhanced BMSCs proliferation at 24 h, 48 h, and 72 h (*P* < 0.01). Thus, antler polypeptide B promoted the growth of BMSCs.

### 3.7. Effects of Different Groups of Antler Polypeptides on the Cell Cycle of BMSCs

The proliferation index reflected the cell cycle process and proliferative ability of BMSCs. The proliferation index of each group was as follows (Figure 4(b)): antler polypeptide B group (38.68%), CCC group (27.74%), blank group (24.70%), antler polypeptide A group (21.39%), and antler polypeptide C group (12.99%). Compared with the blank group, the antler polypeptide B group showed a significantly higher proliferation index, indicating that antler polypeptide B with a molecular weight of 800–1500 had the strongest proliferative effect on BMSCs.

### 3.8. Effect of Antler Polypeptide on the Alkaline Phosphatase Activity of BMSCs

**Figure 5:**
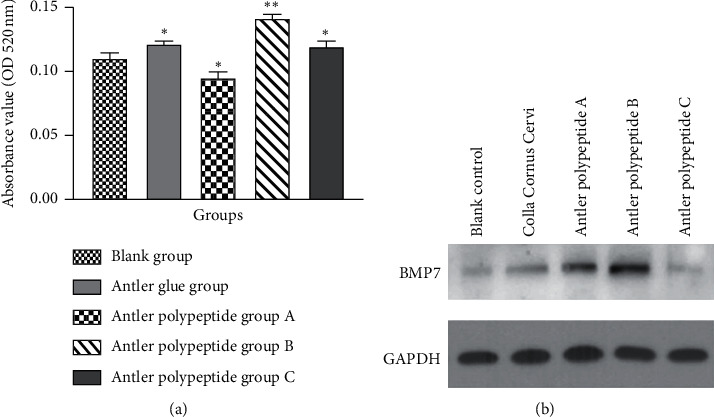
Effect of antler polypeptide on osteogenic differentiation of BMSCs. (a) Antler polypeptide enhanced the ALP activity of BMSCs. (b) Antler polypeptide promoted BMP7 protein expression in BMSCs. ^*∗*^*P* < 0.05, ^*∗∗*^*P* < 0.01, compared with the blank control group.

The activity of ALP was significantly increased in the antler polypeptide B group compared with the blank control group (*P* < 0.001, Figure 5(a)). The CCC group and antler polypeptide groups A and C had significant effects on the ALP activity compared with the blank control group (*P* < 0.05). The results indicate that antler polypeptide enhances the activity of ALP in BMSCs.

### 3.9. Effect of Antler Polypeptide on BMP7 Expression

Western blotting results showed that a BMP7 band was at the expected size. BMP7 protein expression was markedly increased in the antler polypeptide A, B, and CCC group compared with the blank control group (Figure 5(b)). The antler polypeptide B group showed maximum BMP7 expression. The results indicated that antler polypeptide enhanced the BMP7 expression in BMSCs.

## 4. Discussion

The biological activity of antler polypeptide which is separated from antlers has been reported in different diseases [12]. Antler polypeptide plays an important role as an antioxidant and antifatigue. It has been found that antler polypeptide protects MG63 damaged by H_2_O_2_ and effectively improves the survival rate of oxidative damaged MG63 cells. The mechanism may be related to promoting the expression of BMP-2, reducing the content of malondialdehyde (MDA) and Lactate dehydrogenase (LDH), and increasing the activity of superoxide dismutase (SOD) [19].G. Jian proved its good antioxidant activity by determining, in vivo, the superoxide anion scavenging activity, hydroxyl radical (OH) scavenging activity, and the reducing power of red deer horn polypeptide, as well as the SOD activity and MDA content. At the same time, three indexes of the weight-bearing swimming test, blood lactic acid content, and liver glycogen content in mice were determined, which proved that it had an antifatigue effect. In addition, by measuring the carbon clearance phagocytic index and the ability of spleen lymphocyte proliferation in mice, it was found that deer antler peptide could improve the proliferation ability of spleen lymphocytes and enhance immune function in mice [20]. Other studies have shown that pilose antler polypeptide can play a role in the treatment of osteoporosis by preventing bone loss in ovariectomized rats, and its mechanism may be related to promoting the proliferation of chondrocytes and osteoblasts and inhibiting IL-1 and IL-6, which also indicates that pilose antler polypeptide is expected to be an alternative drug for the treatment of postmenopausal osteoporosis [14]. Xie et al. [21] found that pilose antler peptide can partially reverse the formation of osteophyte in the lumbar intervertebral disc of *β*-catenin in mice, improve the infiltration of the facet cartilage, and increase the total area of cartilage by exploring the therapeutic effect of pilose antler peptide on the model of small joint osteoarthritis in mice. It was also found that antler polypeptide could regulate the extracellular matrix by inhibiting cartilage-degrading enzymes MMP13, ADAMTS4, and ADAMTS5, through the Wnt/*ß*-catenin signal pathway [21].

CCC is a kind of traditional Chinese medicine, which contains a large number of proteins, peptides, amino acids, and other components. Related studies have shown that CCC is closely related to the growth of osteocytes [9]. Therefore, this paper made an in-depth study on CCC. The biuret method is usually used to determine the total content of polypeptides in samples. The HPLC method can directly reflect the molecular weight range, composition, and distribution of polypeptides in the sample according to the molecular weight of the reference substances. Xie et al. [22] established a rapid method for the determination of donkey-hide gelatin. In the present study, antler polypeptide was extracted from CCC powder and separated into an antler polypeptide A (<800 Da), B (800–1500 Da), and C (>1500 Da) group according to the molecular weight. The total content of antler polypeptides was detected by the biuret method. At the same time, antler polypeptide was identified through HPLC, and the content of antler polypeptide in group B was significantly higher than that of the other samples. These two methods were used to analyze and detect antler polypeptide in general and in detail, which can better illustrate the composition of antler polypeptide. The effects of antler polypeptide on biological functions of BMSCs were further investigated. Results confirmed that antler polypeptide B with a molecular weight of 800–1500 Da significantly promoted the growth and proliferation of BMSCs, and the optimum concentration was 1.578 × 10^−2^ g/mL. Cell cycle analysis revealed that antler polypeptide B significantly increased the proliferation index of BMSCs. These results suggested that antler polypeptide promoted BMSCs growth and proliferation through promoting the cell cycle process of BMSCs.

The effect of antler polypeptide on osteogenic differentiation of BMSC was further investigated. Results showed that antler polypeptide B markedly enhanced the ALP activity, which is an important indicator of osteogenic differentiation. Furthermore, antler polypeptide B significantly enhanced BMP7 protein expression in BMSCs. BMP7, also known as bone morphogenetic protein 7, is a member of the transforming growth factor *β* family. BMP7 has many biological functions, including regulating cell growth, proliferation, differentiation, apoptosis, and inducing bone formation. A previous study revealed that overexpression of BMP7 significantly promoted the osteogenic differentiation of BMSCs [9]. Kang et al. [23] found that BMP7 enhanced osteogenic differentiation of BMSCs transfected with a BMP7-recombinant-vector. BMP7 enhanced the osteogenic differentiation of human dermal-derived CD105^+^ fibroblast cells through the Smad and MAPK pathways [24]. These researches and our results indicated that BMP7 may associate with osteogenic differentiation of BMSCs promoted by antler polypeptide.

For the mechanism study, it was reported that antler polypeptide may regulate the OPG/RANKL/RANK signaling pathway to prevent osteoporosis [25]. Antler polypeptide enhanced the differentiation of BMSCs and inhibited the growth of osteoclasts by regulating the NF-*κ*B signaling pathway [26]. Antler polypeptide inhibited the activity of IL-1 and IL-6, promoted the proliferation of BMSCs, and inhibited bone loss [21]. Ren et al. [10] reported that pilose antler aqueous extract promoted the proliferation and osteogenic differentiation of bone marrow mesenchymal stem cells by stimulating the BMP-2/Smad1 and 5/Runx2 signaling pathway. Our present study suggested that antler polypeptide promoted proliferation and osteogenic differentiation of BMSCs. In further studies, the molecular mechanisms of antler polypeptide on proliferation and osteogenic differentiation of BMSCs will be investigated.

## 5. Conclusions

In the present study, antler polypeptide was extracted from CCC powder and confirmed through HPLC analysis. Antler polypeptide (molecular weight 800–1500 Da) significantly promoted the proliferation of BMSCs with a proliferation index of 38.68%. Antler polypeptide increased the activity of alkaline phosphatase and enhanced BMP7 protein expression in BMSCs. Our study suggested that antler polypeptide promoted the proliferation and osteogenic differentiation of BMSCs. The present research lays an experimental foundation for the further development and application of antler polypeptide in medicine.

## Figures and Tables

**Table 1:** The peptide content of A, B, and C samples determined by the biuret method.

Samples	Volume (mL)	Average absorbance	Concentration (mg/mL)	Antler polypeptide content (mg/mL)
A	0.7	0.291	6.319	0.602
B	0.8	0.331	7.181	8.976
C	0.2	0.358	7.776	38.88

*Note*. Antler polypeptide A (molecular weight <800 Da) sample was concentrated 15 times.

**Table 2:** The peak area results of different samples detected by HPLC (*n* = 3).

Group	Eluted time/min	Peak area
A	—	—
B	14.460	933.80927
C	14.907	20.30075

*Note*. The peptide peak of different samples with the molecular weight 800–1500 Da eluted at 14.279∼15.351 min.

## Data Availability

Data sharing is not applicable to this article as no datasets were generated or analyzed during the current study.

## Abbreviations

ALP: Alkaline phosphatase
ANFH: Avascular necrosis of the femoral head
BMSCs: Bone marrow-derived mesenchymal stem cells
CCC: Colla Cornus Cervi
DMEM-LG: Dulbecco's modified Eagle's medium-low glucose
HPLC: High-performance liquid chromatography
PBS: Phosphate buffered saline solution.
